# Impairments, activity limitations and participation restrictions: Prevalence and associations among persons living with HIV/AIDS in British Columbia

**DOI:** 10.1186/1477-7525-2-46

**Published:** 2004-09-06

**Authors:** Melanie Rusch, Stephanie Nixon, Arn Schilder, Paula Braitstein, Keith Chan, Robert S Hogg

**Affiliations:** 1 Department of Population Health, British Columbia Centre for Excellence in HIV/AIDS, St. Paul's Hospital, Vancouver, Canada; 2 Department of Health Care and Epidemiology, University of British Columbia, Vancouver, Canada; 3 Department of Physical Therapy, University of Toronto, Toronto, Canada; 4 British Columbia Persons with AIDS (BCPWA) Society, Vancouver, Canada

**Keywords:** disablement, impairments, activity limitations, participation restrictions, disability, HIV/AIDS

## Abstract

**Background:**

To measure the prevalence of and associations among impairments, activity limitations and participation restrictions in persons living with HIV in British Columbia to inform support and care programs, policy and research.

**Methods:**

A cross-sectional population-based sample of persons living with HIV in British Columbia was obtained through an anonymous survey sent to members of the British Columbia Persons With AIDS Society. The survey addressed the experience of physical and mental impairments, and the experience and level of activity limitations and participation restrictions. Associations were measured in three ways: 1) impact of types of impairment on social restriction; 2) impact of specific limitations on social restriction; and 3) independent association of overall impairments and limitations on restriction levels. Logistic regression was used to measure associations with social restriction, while ordinal logistic regression was used to measure associations with a three-category measure of restriction level.

**Results:**

The survey was returned by 762 (50.5%) of the BCPWA participants. Over ninety percent of the population experienced one or more impairments, with one-third reporting over ten. Prevalence of activity limitations and participation restrictions was 80.4% and 93.2%, respectively. The presence of social restrictions was most closely associated with mental function impairments (OR: 7.0 for impairment vs. no impairment; 95% CI: 4.7 – 10.4). All limitations were associated with social restriction. Among those with ≤ 200 CD4 cells/mm3, odds of being at a higher restriction level were lower among those on antiretrovirals (OR: 0.3 for antiretrovirals vs. no antiretrovirals; 95% CI: 0.1–0.9), while odds of higher restriction were increased with higher limitation (OR: 3.6 for limitation score of 1–5 vs. no limitation, 95%CI: 0.9–14.2; OR: 24.7 for limitation score > 5 vs. no limitation, 95%CI: 4.9–125.0). Among those with > 200 CD4 cells/mm3, the odds of higher restriction were increased with higher limitation (OR: 2.7 for limitation score of 1–5 vs. no limitation, 95%CI: 1.4–5.1; OR: 8.6 for limitation score > 5 vs. no limitation, 95%CI: 3.9–18.8), as well as by additional number of impairments (OR:1.2 for every additional impairment; 95% CI:1.1–1.3).

**Conclusions:**

This population-based sample of people living with HIV has been experiencing extremely high rates of impairments, activity limitations and participation restrictions. Furthermore, the complex inter-relationships identified amongst the levels reveal lessons for programming, policy and research in terms of the factors that contribute most to a higher quality of life.

## Background

For most people who are able to access and tolerate highly active antiretroviral therapy (HAART), HIV/AIDS has become a chronic condition characterized by cycles of illness and wellness. People live longer lives, but with physical, psychological and social challenges that affect quality of life [1-3]. Evidence of this phenomenon may be found in qualitative studies describing the ways in which improved health has also brought about different and unforeseen social, psychological and physical challenges for many people who had previously been facing end-stage disease.

For instance, Brashers et al (1999) identified four categories of "uncertainties" resulting from the experience of "revival" brought about by HAART, including (a) renegotiating feelings of hope and future orientation in the face of questionable durability of immune restoration; (b) fear about social roles and identities, in the transition from a person who is dying to a person living with a chronic illness; (c) concerns with interpersonal relations, including the potential of stigmatizing reactions from employers and co-workers; and, (d) reconsidering the quality of their lives, captured in this quote from one participant, 'The good news is you're going to live, the bad news is you're not going to enjoy the rest of your life' [1].

Sowell et al. (1998) used in-depth interviews to explore the psychological changes and care delivery issues experienced by HIV-positive men who were facing end-stage disease but had experienced dramatic physical improvements [4]. Key findings included themes around protease inhibitors as a reprieve from death, shifting perspectives on roles and relationships, and a renewed need for advocacy related to care, treatment and support. Others have examined particular aspects of living with HIV in the post-HAART era, such as challenges related to income and employment [5]. Along with qualitative literature, the HIV communities themselves have responded with a wave of community-based studies, publications and programming to address challenges related to living with the ups and downs of life on combination therapies [6-9].

Quantitative studies exploring the life-and health-related consequences of living with HIV are limited. An exception is the HIV Cost and Services Utilization Survey in the United States, which described physical and social role restrictions in a nationally representative sample [10]; however, no similar work exists in Canada. The American study was undertaken during the early years of HAART, and so the majority of participants were not yet on protease inhibitors. As such, there is a gap in the literature in terms of studies that systematically quantify the prevalence of life-and health-related challenges associated with living with HIV since the advent of HAART.

The International Classification of Functioning, Disability and Health (WHO, 2001) offers a useful framework for studying disablement and health-related consequences of disease based on the following three concepts: impairments, activity limitations and participation restrictions [11]. Impairments are understood to be problems with physiological functioning or anatomical (e.g., organs, limbs) structure of the body. Activity limitations are defined as difficulties in executing a task or action. Finally, participation restrictions are problems relating to involvement in life situations. This classification system and its precursor, the International Classification of Impairments, Disabilities and Handicaps (WHO, 1980), have been used to frame a plethora of studies on a diverse array of diseases and conditions [12-15]. Furthermore, this framework has been used to conceptualize HIV [16], and informs the policy, research and advocacy work of organizations such as the Canadian Working Group on HIV and Rehabilitation [17].

This article addresses this gap in the literature by reporting on the results of a quantitative investigation into the prevalence of and associations among impairments, activity limitations and participation restrictions experienced by people living with HIV in British Columbia.

## Methods

### Data sources

Individuals living with HIV were involved in all stages of this project, from identification of the research question to data collection and analysis. A lead partner was the British Columbia Persons With AIDS Society (BCPWA), an organization of more than 3,600 HIV positive individuals living in British Columbia, which was created to provide support, information and advocacy for its members.

From May to September of 2002, the BCPWA in conjunction with the British Columbia Centre for Excellence in HIV/AIDS conducted a survey of HIV positive individuals living in British Columbia. The anonymous self-administered questionnaire was mailed to the 1508 HIV positive individuals registered with the BCPWA who had consented to receive mailings.

### Definition of disability

A section of the survey on diagnosed conditions asked participants to indicate if a doctor had ever in their lifetime diagnosed them with any conditions from a list of thirteen, including depression, schizophrenia, bipolar disorder and post-traumatic stress disorder, as well as a space to indicate any diagnoses that was not present in the list.

Participants identified their experiences during the past month using check-lists of impairments, activity limitations and participation restrictions that included space to identify unlisted items.

Participants were asked: "Within the last month have you experienced any of the following..." after which they were able to check off symptoms from a list of twenty-two, including a space for unlisted items. The list of impairments was categorized into mental, internal system, sensory and neuromusculoskeletal groups based on the International Classification of Functioning, Disability and Health [12]. Mental impairments included reduced libido, poor concentration, poor appetite, chronic fatigue, decreased endurance, decreased memory, impaired cognition and aphasia. Internal impairments included diarrhea, gastric reflux, shortness of breath, constipation, wasting, weakness, vomiting and incontinence. Sensory impairments included headaches, altered sensations, nausea, mouth pain and decreased vision. Neuromusculoskeletal impairments included altered muscle tone, stiff joints, seizures, hemiparesis and paraparesis. This section was followed by a question which asked participants how much HIV-related pain they had experienced in the past month, with categorical options including none, a little bit, mild or infrequent, moderate, severe or persistent and don't know. Participants were also asked to pinpoint the location(s) of their HIV related pain.

Activity limitations were addressed by asking the participants " [h]ow well can you manage these typical daily activities?" with an indication to circle the response which best describes their experience in the past month. A fifteen-item list including ability to walk one block, eat, shower, and dress followed. For each item, participants indicated whether they were (a) completely able, (b) somewhat limited or (c) unable to perform the activity. Overall prevalence of activity limitations was calculated by including anyone indicating (b) or (c) for any one of the fifteen items.

In the same way, participants were asked " [h]as your health limited your usual [role/participation]" in any of a number of categorical activities and functions. Participants were indicated to choose the response that came closest to the way they had been feeling during the past month. A ten-item list was used to assess levels of restriction in social, student, and cultural roles. Participants indicated whether they were (a) not limited, (b) somewhat limited or (c) very limited with respect to their ability to function in these roles. Overall prevalence of participation restrictions was calculated by including anyone indicating (b) or (c) for any one of the ten items.

### Statistical analysis

Rates of impairments, activity limitations and participation restrictions among the participants were compared across three categories of CD4 cell counts (≤ 200 cells/mm3, 201 to 500 cells/mm3 and > 500 cells/mm3) using a chi-squared test for categorical variables and the Kruskal-Wallis test for continuous variables. Bonferroni corrections for multiple comparisons were done for each item and those which remained significant are indicated in bold.

To test the hypothesis that social role restrictions would be more strongly associated with mental function impairments and personal care and mobility limitations, a series of logistic regression models were tested with each category of impairment and limitation. A dichotomous outcome was used, collapsing "somewhat" and "very much" social role restriction into any social restriction. Likewise, specific activity limitations were dichotomized into "no limitations" vs. "some effort" required or "unable" to accomplish the activity. Associations of social restriction with impairment categories and specific activity limitations were examined univariately and in adjusted models accounting for age, sex, income, depression, pain, risk category (men who have sex with men, injecting drug users, heterosexual contact, combination) and number of symptoms for activity limitation models.

A scoring system was then used to develop categories of activity limitation and participation restriction. If a participant indicated an activity limitation item at the highest level ("unable" to accomplish) or a participatory role restriction at the highest level ("very much" restricted), two points were received, while participants indicating an activity limitation item at moderate level (requiring "effort" to accomplish) or a participation restriction item at a moderate level ("somewhat" restricted), one point was received. Overall scores for participation restriction and activity limitation were therefore dependent on both the severity and total number of challenges in activities or participatory roles.

The participation restriction score, with an overall maximum of 20, was then categorized into three levels: 0 to 5 points, 6 to 10 points and > 10 points, based on the population distribution of the score. Likewise, the activity limitation score, with an overall maximum of 28, was also categorized based on distribution as follows: 0, 1 to 5 points, and > 5 points. The higher the score, the greater the disablement.

An overall model examined the associations of increasing participation restriction level with number of impairments and activity limitation scores, testing the hypothesis that impairments may account for some of the associations seen between activity limitations and participation restrictions, but that both of the former would have independent associations with the latter. Ordinal logistic regression was implemented, using the three-level participation restriction outcome and testing number of symptoms, categorical limited activity score, pain and mental diagnoses as explanatory variables. All models were stratified on CD4 levels, with separate models built for individuals with counts below 200 cells/mm3, and adjusted for age, gender, employment, years since diagnosis and risk category.

## Results

### Population characteristics

Of the 762 people living with HIV who completed the survey, 614 provided information about their CD4 levels and were included in this analysis. The population answering the BCPWA survey was comprised mainly of white (88.7%), sexual-minority males (76.6%) between the ages of 30 to 49 (63.9%). The 148 respondents who were not included in the analysis because they did not provide CD4 information were in a lower income bracket (42.5% vs 19.9%; p-value < 0.001), were more likely to be current IDUs (11.3% vs 4.3%; p-value < 0.001) and more likely to be First Nations/Inuit/Metis (17.6% vs. 6.5%; p-value < 0.001).

A comparison of all BCPWA members who received the survey and the subset who responded found a similar distribution of age and a similar proportion identifying as Aboriginal (7.1% vs. 8.7%). The proportion of females was higher among the total BCPWA population than among the subset of respondents (13.5% vs.10.2%; p = 0.001).

### Prevalence of impairments, activity limitations and participation restrictions

Table 1 describes levels of diagnoses, impairments, activity limitations and participation restrictions among participants. Mental health diagnoses were reported by 62.9% (N = 479) of the participants. The most prevalent diagnosis was depression with an overall prevalence of 58.1%. Among those listing one or more diagnoses, 92.5% experienced depression as one of their diagnoses. While the overall number of participants with depression appeared lower among those with CD4 ≤ 200 cells/ml, the percent of those listing depression out of those with any diagnosis remained close to 92.5% across all strata.

**Table 1:** Prevalence of diagnosed conditions, impairments and pain, activity limitations and participation restrictions experienced by BCPWA participants by CD4 cell counts

	**CD4 < 200**	**CD4 201 to 500**	**CD4 > 500**	**p-value**
**Diagnosed conditions**				
Depression	64 (52.0)	183 (59.2)	110 (61.5)	0.238
General Anxiety	11 (8.9)	34 (11.0)	14 (7.8)	0.488
Post traumatic Stress	6 (4.9)	18 (5.8)	13 (7.3)	0.677
Panic Disorder	8 (6.5)	35 (11.4)	12 (6.7)	0.124
**Median number of impairments (IQR)**	**9 (5, 13)**	**7 (2.5, 12)**	**7 (3, 12)**	**0.006**
**% With any impairment**	120 (97.6)	285 (92.5)	161 (89.9)	0.041
**Pain**				
None	25 (20.7)	82 (29.4)	48 (28.4)	0.079
Little/mild	35 (28.9)	89 (31.9)	62 (36.7)	
Moderate/severe	61 (50.4)	108 (38.7)	59 (34.9)	
**Median number of activity limitations (IQR)**	3 (1, 7)	3 (1, 7)	2 (1, 5)	0.015
**% With any Activity Limitation**	108 (87.8)	236 (77.4)	137 (76.5)	0.031
**Median number of Participation Restrictions (IQR)**	7 (4, 9)	7 (3, 9)	7 (3, 9)	0.251
**% With any Participation Restrictions**	121 (98.4)	278 (91.5)	161 (89.9)	0.017

**Bold **print indicates comparison that remained significant at the p = 0.016 level after Bonferroni correction for multiple comparisons.

The presence of multiple impairments among the participants was also high, with a median of 7 (3,12) impairments and approximately one third of the participants experiencing more than ten impairments. At least one impairment was reported by 91.5% (N = 697). There was a significant difference in the distribution of impairments across CD4 categories, which remained after Bonferroni correction (CD4 ≤ 200 cells/ml vs CD4 > 500 cells/ml, p-value= 0.002; CD4 ≤ 200 cells/ml vs CD4 between 200 and 500 cells/ml, p-value = 0.017). Mental impairment was reported by 78.2% (N = 596), sensory impairment by 71.9% (N = 548), neuromuscular impairment by 49.5% (N = 377), and internal impairment by 81.0% (N = 617) of the participants.

Pain was reported by over half of the participants, and by over three quarters of the participants with CD4 ≤ 200 cells/ml. Approximately one-third reported little or mild pain and 37.1% reported moderate or severe pain. For participants with lower CD4 counts, more people reported moderate and severe pain (50.4% vs. 38.7% vs. 34.9%; p-value 0.08), although comparisons of each CD4 category to the others showed no significant differences.

Activity limitations were reported by 80.6% (N = 607) of the participants. The median number of activity limitations reported by an individual was 3 (1, 7). Six hundred and ninety-nine individuals (93.2%) reported some level of participation restriction. The median number of participatory roles in which individuals felt somewhat or highly restricted was 7 (3, 9). Although distributions of activity limitations and participation restrictions were significantly different, adjustment for multiple comparisons across the CD4 categories resulted in no significant difference in prevalence.

**Figure 1:**
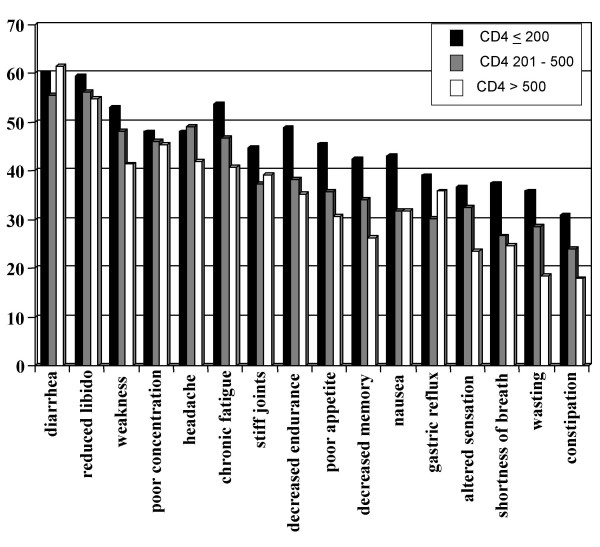
Prevalence of specific impairments for participants with CD4 counts ≤ 200 cells/mm3 (speckled bars), 201 to 500 cells/mm3 (downward diagonally-striped bars) and > 500 cells/mm3 (horizontally-striped bars). Significant p-value from chi-square test across CD4 categories.

**Figure 2:**
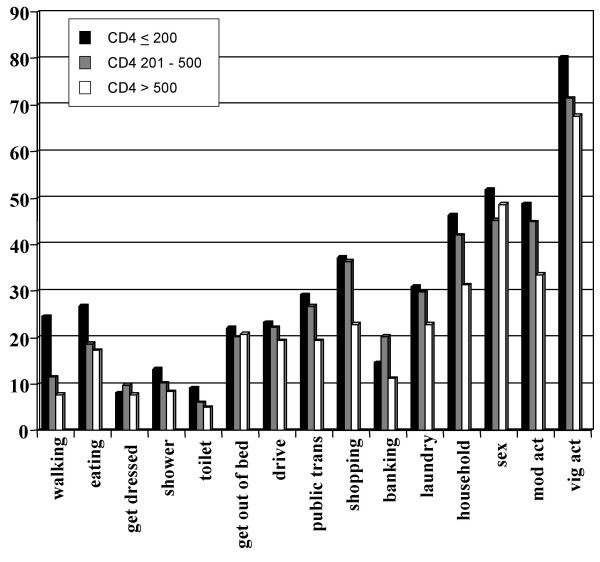
Prevalence of specific activity limitations for participants with CD4 counts ≤ 200 cells/mm3 (speckled bars), 201 to 500 cells/mm3 (downward diagonally-striped bars) and > 500 cells/mm3 (horizontally-striped bars). Significant p-value from chi-square test across CD4 categories.

**Figure 3:**
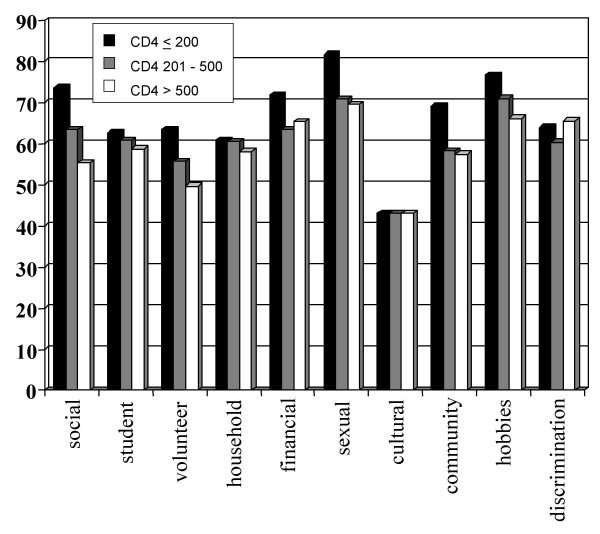
Prevalence of specific participation restrictions for participants with CD4 counts ≤ 200 cells/mm3 (speckled bars), 201 to 500 cells/mm3 (downward diagonally-striped bars) and > 500 cells/mm3 (horizontally-striped bars). Significant p-value from chi-square test across CD4 categories.

Figures 1,2,3 summarize the prevalence of impairments, activity limitations and participation restrictions, respectively. The most prevalent impairments experienced by participants included diarrhea (57.1%), reduced libido (55.8%), general weakness (48.2%), poor concentration (47.0%), headaches (46.9%) and chronic fatigue (46.6%). Vigorous and moderate activities, sexual activities and household chores were the most frequently reported limitations. The level of participation restrictions was high for all CD4 categories, with sexual roles, student/employee roles and financial roles being the most prevalent.

### Univariate associations of impairments and activity limitations on social role restrictions

Table 2 describes the univariate odds ratios for presence of social role restriction (yes vs. no) based on impairment categories and type of activity limitation. All impairments and activity limitations were significantly associated with social role restriction. Social role restriction was most strongly associated with limitations in using the toilet, (OR: 18.5 for toilet difficulties vs. no toilet difficulties; 95%CI: 4.5 – 76.3), followed by banking, (OR: 11.3 for banking difficulties vs. no banking difficulties; 95%CI: 5.4 – 23.5). Social role restriction had the weakest association with getting out of bed, (OR: 3.6 for difficulties getting out of bed vs. no difficulties; 95%CI: 2.3 – 5.6). With respect to impairment categories, social role restriction was most strongly associated with mental impairments (OR 7.0 for mental impairments vs. no mental impairments; 95% CI 4.7–10.4) although the other three impairment categories had odds ratios higher than four.

**Table 2:** Univariate and adjusted odds ratios for social role restriction given each activity limitation and prevalence of these limitations in this population

**Activity**	**Prevalence (%)**	**Odds Ratio (95% CI)**	Adjusted Odds Ratio (95% CI)*	
			≤ 200 cells/ml	> 200 cells/ml
			
Getting dressed	8.9 (54)	4.03 (2.03 – 8.00)	7.90** (0.45 – 137)	1.60 (0.47 – 5.44)
Using the toilet	6.3 (38)	18.47 (4.47 – 76.3)	9.14** (0.52 – 159)	37.7** (2.29 – 620)
Showering	10.2 (62)	6.62 (3.15 – 13.91)	3.38 (0.41 – 28)	2.30 (0.57 – 9.18)
Walking one block	13.2 (80)	5.33 (2.86 – 9.91)	3.33 (0.54 – 20)	3.40 (0.72 – 16)
Banking	16.4 (99)	11.27 (5.42 – 23.45)	3.78 (0.33 – 42)	**3.30 (1.09 – 10)**
Getting out of bed	20.8 (125)	3.63 (2.34 – 5.63)	1.15 (0.31 – 4.14)	1.89 (0.79 – 4.54)
Driving	21.5 (121)	3.51 (2.25 – 5.47)	1.59 (0.45 – 5.49)	1.93 (0.87 – 4.31)
Eating	20.1 (122)	4.66 (2.89 – 7.53)	0.87 (0.26 – 2.94)	**3.17 (1.07 – 9.37)**
Public Transportation	25.2 (148)	6.75 (4.19 – 10.86)	4.36 (0.91 – 21)	**3.29 (1.32 – 8.20)**
Laundry	28.1 (171)	7.53 (4.73 – 11.98)	**8.41 (1.32 – 54)**	**3.26 (1.41 – 7.52)**
Groceries	32.6 (198)	8.43 (5.38 – 13.21)	**3.97 (1.21 – 13)**	**2.97 (1.37 – 6.43)**
Household chores	39.6 (241)	6.89 (4.72 – 10.06)	**5.12 (1.62 – 16.2)**	**3.11 (1.59 – 6.10)**
Moderate activity	42.4 (258)	5.87 (4.11 – 8.37)	2.10 (0.76 – 5.77)	**3.10 (1.62 – 5.93)**
Sexual activity	46.6 (283)	5.33 (3.81 – 7.47)	2.56 (1.00 – 6.57)	**2.06 (1.16 – 3.68)**
Vigorous activity	71.9 (437)	5.09 (3.61 – 7.19)	2.69 (0.97 – 7.48)	**2.60 (1.37 – 4.96)**
**Impairment Category**				
Mental functioning	78.7 (481)	7.02 (4.73 – 10.4)	**18.71 (2.31 – 151)**	**4.32 (2.20 – 8.51)**
Neuro-musculoskeletal functioning	49.3 (301)	4.12 (2.98 – 5.69)	1.77 (0.67 – 4.68)	**1.76 (1.03 – 3.00)**
Sensory functioning	72.3 (442)	4.12 (2.94 – 5.78)	0.85 (0.27 – 2.65)	**2.17 (1.20 – 3.93)**
Internal functioning	81.4 (500)	4.15 (2.82 – 6.12)	2.48 (0.69 – 8.91)	1.84 (0.96 – 3.51)

*Adjusted for age, gender, income, number of impairments (for activity limitation models), pain, risk category and doctor-diagnosed depression

**Small sample size; analysis stratified on CD4 but not adjusted due to zero cells.

Adjusted odds ratios stratified by CD4 counts remained significant for getting groceries, doing laundry, household chores, and mental functioning, regardless of CD4 levels, although the estimates were higher for participants with counts under 200 cells/mm3. For those with CD4 counts above 200 cells/mm3, difficulties with eating, public transportation, moderate or vigorous activities, sexual activities, neuromuscular functioning and sensory functioning also remained significantly associated with social restrictions. Adjusted odds ratios for using the toilet and getting dressed were unable to be estimated as these were co-linear with the outcome. Stratified, unadjusted estimates of 9-fold and 37-fold increases in social restriction were seen with limitations in toileting.

Stratification by CD4 levels indicated a general effect modification across activity limitations and impairment categories, with greater, although more unstable, associations with social restrictions being found among participants with < 200 cells/mm3.

### Multivariate associations of impairments and activity limitations with participation restriction levels

Table 3 describes the ordinal logistic regression model examining associations with a three-category measure of participation restriction level, stratified by CD4 cell counts. Among those with CD4 counts under 200 cells/mm3, being in a higher category of participation restriction was strongly associated with having activity limitation scores above ten, and was marginally inversely associated with being on antiretrovirals. Increasing number of impairments did not show any significant association.

**Table 3:** Ordinal logistic regression estimating the probability of being in a higher category of the three level participation restriction score based on levels of impairment, limited activity scores and pain.

	**CD4 ≤ 200**	
	
	OR*	**95% CI**
**Limited Activity score**		
None	1	
1–5	3.58	0.91 – 14.2
> 5	24.7	4.85 – 125
**Number of impairments**	1.01	0.94 – 1.12
**Antiretroviral use**	0.28	0.08 – 0.93

	CD4 > 200	
	
	OR*	**95% CI**

**Limited Activity score**		
None	1	
1–5	2.67	1.40 – 5.12
> 5	8.56	3.90 – 18.8
**Number of impairments**	1.19	1.12 – 1.25
**Pain**		
None	1	
Some/mild	1.31	0.71 – 2.44
Mod/severe	1.78	0.85 – 3.75
**Antiretroviral use**	1.39	0.83 – 2.35

*adjusted for age, gender, employment, education, years since diagnosis and risk category

Among participants with CD4 counts above 200 cells/mm3, being in a higher category of participation restriction was associated with increasing levels of limited activity [(OR: 2.7 for limited activity scores of 4–10 vs. scores < 4; 95%CI: 1.4–5.1) and (OR: 8.6 for limited activity scores > 10 vs. scores < 4; 95%CI: 3.9–18.8)]. A higher participation restriction category was also significantly associated with increasing number of impairments, with a 19% increase in the odds with additional impairment. Increased participation restriction level was only marginally significantly associated with moderate or severe pain; however, point estimates for the pain categories suggested a dose response relationship, as did the inclusion of pain as a continuous variable (p-value 0.066).

## Discussion

This study has demonstrated that a population-based sample of people living with HIV in British Columbia have been experiencing strikingly high levels of depression, body impairments, activity limitations and participation restrictions. The latter two categories were higher among this population than a national survey of HIV positive persons in the United States[10]. However, the American study was conducted prior to HAART availability, underscoring the importance of examining quality of life issues faced in the post-HAART era. In a study examining similar concepts of activity limitation among cancer patients, the percent experiencing any difficulties ranged from 18.0 to 70.0%, depending on the type of cancer, but was only 30.0% overall [18]. Another study of cancer survivors found a similar prevalence to that seen in the present study (80.0%) when including all ambulatory difficulties, not just activities of daily living [19]. The elevated levels of limitation among the BCPWA population were also emphasized in a comparison with the general population and with those identifying as suffering from a chronic illness, where the least difference showed a five-fold increase [20].

The level of depression among this population was extremely high. Nearly 60.0% of the participants reported ever having been diagnosed with depression by a doctor. Levels of depression among HIV positive persons reported in the literature range from 5.0% to 40.0%, although among HIV positive women, 60.0% prevalence has been reported [21,22]. Depression is generally found to be higher, regardless of HIV status, among women and men who have sex with men [21]. Studies conducted among MSM have found prevalence of major depression to range from 23.0 to 37.0%, while Aboriginal populations in general, and Aboriginal MSM in particular, have been shown to have higher depression scores [23-25]. Likewise, depression among IDU populations has been seen to be as high as 47.0% [24]. Some study scales may capture current depression but may miss the experience of people with recurrent episodes who feel well at the time of testing. The high level of depression recorded in this study may be the result of a large percentage of men who have sex with men in the sample as well as the survey's ability to capture more cumulative measures of depression. The high prevalence may be due in part to the self-report of the diagnosis as well, which may result in recall bias and increased reporting of non-diagnosed depression. Regardless, this common experience of depression demands consideration by researchers, policy-makers and care providers concerned with the quality of life of people living with HIV.

The prevalence of impairments was also high, with diarrhea at the top of the list, followed by problems with fatigue and endurance. Furthermore, challenges with daily activities and social roles were extremely common, at greater than 80.0 and 90.0%, respectively. The high proportion of individuals experiencing impairments, activity limitations and participation restrictions sheds light on the spectrum of challenges related to living with HIV. Even among those with relatively high CD4 counts, the impact of HIV on disability and health is not trivial.

Of note, the differences experienced between people according to categories of CD4 levels were less and less apparent going from impairments (problems at the level of organ or body part) to activity limitations to participation restrictions (problems with social roles). This draws attention to the variety of influences affecting a person's ability to perform daily tasks and participate in regular societal roles above and beyond his/her clinical measures of disease status.

All types of activity limitation were associated with the experiencing of social role restrictions. After accounting for impairments, depression and pain levels, there remained significant associations between household upkeep, including laundry and groceries, and social role participation. Although it was hypothesized that personal care issues would have stronger associations, this was not the case. This may be because the severity of personal care limitations (dressing, eating, showering) is such that among those experiencing these limitations, the presence of pain or numerous impairments overshadows any independent association between the limitation and social restriction.

Household chores, getting groceries and doing laundry as well as moderate and vigorous activities had significant associations with social role restrictions and had the highest prevalence in this population. Therefore, interventions that target these types of limitations may provide the most benefit at a population level. Whether or not these interventions would have any impact on an individual's feelings of participatory restriction remains to be seen; however, coordinating these types of simple interventions might offer contact with people in need of social support.

Mental impairments were the most prevalent of the four impairment categories and were found to have a significant association with participation restrictions. These results mirror a study describing disability among a national sample of people living with HIV in the United States which reported a correlation between general fatigue and increased limitations in both physical and role functions [10]. Other reports have also found relationships among neuropsychological performance, depression, stress levels and perceived disability [26]. It is suggested that increased social support networks can result in improved mental health, which may indicate that the association between the presence of mental impairment and the ability to interact in social and community roles is not unidirectional.

The adjusted models (Table 3) indicate that both impairments and activity limitations remain associated with participation restrictions independent of one another for people with high CD4 counts. The use of antiretrovirals among those with low CD4 counts is associated with lower participation restriction levels. Since this cannot be accounted for through a lessening of impairments or limitations among those on antiretrovirals, it is more likely a reflection of the type of support and interaction with the health care system among those who are able to access antiretrovirals.

### Limitations of the study

Limitations of the study include the somewhat homogeneous nature of the participants, which affects the generalizability of these findings to other populations. The participants were mainly white, sexual-minority males with moderate yearly incomes and stable housing. The under-representation of people who are homeless, injection drug users, female and Aboriginals becomes apparent when comparing the low proportions seen amongst the BCPWA membership to the higher proportions seen in incident cases reported by the British Columbia Centre for Disease Control [27].

The survey was sent to BCPWA members consenting to receive mail. Individuals who did not consent were more likely to reside in the Greater Vancouver region, suggesting a greater geographical representation from outside of this urban area. Non-consenting BCPWA members were also more likely to be female (15.8% vs 11.9%) and more likely to be First Nations, Inuit or Metis (27.1% vs 8.4%). Furthermore, because the survey was anonymous and self-reported, there are issues with missing data and incomplete records. For example, almost 20.0% of the sample, again representing a high proportion of women and First Nations, were excluded because of missing CD4 information. While the exclusion of this population may have affected the power and generalizability of the study, one may argue that challenges reported in this study may be an underestimation of the restrictions in this population due to compounding social inequity issues.

Lastly, there are limitations in the nature of self-reported diagnoses. Participants may have trouble recalling the presence or absence of impairments, limitations or restrictions over the past month. Although there was no direct incentive, participants may be biased towards increased reporting of problems as they may feel that this would be beneficial for program funding and support.

Despite these limitations, this survey represents a large provincial sample and is one of few attempts to collect information from a population-based sample on this scale. Furthermore, this is one of the first studies to systematically quantify levels of disablement among persons living with HIV.

## Conclusions

This study revealed a strikingly high prevalence of impairments, activity limitations and participation restrictions among a population-based sample of people living with HIV in British Columbia. The complicated interplay among these categories requires further study, but it is clear that interventions designed to help overcome activity limitations and social support programs are required, especially those addressing mental impairments and depression. While impairments and limitations are not always reversible, innovative programs that help people living with HIV address these challenges may help to decrease the subsequent high rates of participatory restrictions experienced. Antiretroviral treatments have enabled the prolongation of the lives of people who are HIV-infected; now we need to give due attention to optimizing the quality of these extended lives.

## Authors' Contributions

MR and KC carried out the statistical analyses; SN and AS participated in the design of the study and the development of the study instrument; PB participated in the conceptualization of the study and the interpretation of the results; RH participated in the conceptualization and design of the study. All authors read and approved the final manuscript.
